# The PRIMERO birth cohort: Design and baseline characteristics

**DOI:** 10.1016/j.jacig.2025.100470

**Published:** 2025-04-11

**Authors:** Jonathan I. Witonsky, Jennifer R. Elhawary, Celeste Eng, Sam S. Oh, Sandra Salazar, Maria G. Contreras, Vivian Medina, Elizabeth A. Secor, Priscilla Zhang, Jamie L. Everman, Ana Fairbanks-Mahnke, Elmar Pruesse, Satria P. Sajuthi, Chih-Hao Chang, Tsunami Rosado Guerrero, Keyshla Canales Fuentes, Natalie Lopez, Chris Angely Montañez-López, Emily Vazquez Morales, Nicole Vazquez Morales, Richeliz Alfonso Otero, Raymarie Colon Rivera, Leysha Rodriguez, Gabriela Vazquez, Donglei Hu, Scott Huntsman, Nathan D. Jackson, Yingchun Li, Andrew Morin, Natalie A. Nieves, Cydney Rios, Gonzalo Serrano, Blake J.M. Williams, Elad Ziv, Camille M. Moore, Dean Sheppard, Esteban González Burchard, Max A. Seibold, Jose R. Rodríguez-Santana

**Affiliations:** a Department of Pediatrics, University of California, San Francisco, Calif; b Department of Medicine, University of California, San Francisco, Calif; c Centro de Neumología Pediátrica, San Juan, Puerto Rico; d Center for Genes, Environment & Health, National Jewish Health, Denver, Colo; e Department of Pediatrics and Division of Pulmonary Sciences and Critical Care Medicine, Department of Medicine, University of Colorado, Aurora, Colo

**Keywords:** Childhood asthma, respiratory illness, birth cohort, gene–environment interactions, transcriptomics, viral pathogen detection, health disparities, Puerto Rico

## Abstract

**Background:**

Although early-life respiratory illnesses (RIs) are linked to childhood asthma, it is unclear whether children are predisposed to both conditions or if RIs induce alterations that lead to asthma. Puerto Rican children, who bear a disproportionate burden of early-life RIs and asthma, are an important population for studying this relationship.

**Objective:**

We sought to describe the design and baseline characteristics of the Puerto Rican Infant Metagenomic and Epidemiologic Study of Respiratory Outcomes (PRIMERO) birth cohort.

**Methods:**

PRIMERO is designed to examine the role of respiratory viruses on the development of RIs and asthma. Pregnant women were recruited at Hospital Interamericano de Medicina Avanzada–San Pablo in Caguas, Puerto Rico. Questionnaires at birth and annual follow-ups gather clinical, social, and environmental data. Collected samples include postterm maternal blood; infant cord blood; the child’s blood at year 2; and the child’s nasal airway epithelium at birth, during RIs over the first 2 years, and annually until age 5.

**Results:**

We enrolled 2,100 mother–child dyads into the PRIMERO study between February 2020 and June 2023, representing 59% of births at Hospital Interamericano de Medicina Avanzada. As of April 29, 2024, 2,069 participants remain active, with high rates of biospecimen collection and annual visit participation. Illness surveillance detected 6,076 RIs, with 38.4% involving the lower respiratory tract.

**Conclusion:**

The PRIMERO birth cohort study, with its comprehensive data on viral exposures, respiratory outcomes, and airway molecular phenotypes in a high-risk population of Puerto Rican children, is uniquely positioned to address long-standing questions about the early-life determinants and mechanisms underlying virus-related asthma development.

Asthma is the most common chronic childhood condition, disproportionately affecting racially and ethnically minoritized children.^1^ Asthma is over 40% more prevalent among Black and Latino children compared to their non-Hispanic White counterparts, and Puerto Rican children face a 3-fold higher likelihood of having asthma and an over 5-fold higher risk of asthma-related mortality.^2^, ^3^, ^4^ Asthma development is influenced by poorly understood and complex gene–environment interactions.^5^, ^6^, ^7^, ^8^, ^9^, ^10^, ^11^, ^12^, ^13^ A key factor perpetuating asthma inequities among Puerto Rican children is their increased early-life exposure to environmental risk factors, especially respiratory viruses associated with asthma development, which are ubiquitous and endemic in Puerto Rico.^13^, ^14^, ^15^, ^16^ Irrespective of race or ethnicity, viral infections causing lower respiratory tract symptoms in the first 2 years of life are commonly linked to the subsequent development of recurrent wheezing and childhood asthma.^17^^,^^18^ In particular, infants who had bronchiolitis or wheezing induced by respiratory syncytial virus (RSV) are 2.6 times more likely to develop asthma by age 6.^14^ Furthermore, children who remain free from RSV infection by 1 year of age have a 26% lower risk of developing asthma before age 5 compared to those infected within the first year.^19^ Similarly, children with early-life wheezing illnesses caused by human rhinoviruses (HRVs) have a 9.8 times higher risk of developing asthma by age 6.^14^

Important questions remain about the early-life viral drivers of asthma, including (1) the magnitude of risk for asthma conferred by HRV versus RSV infections and whether infection with other common viruses play significant roles in driving asthma risk, and (2) whether these virus species independently contribute to asthma risk or if their effects are influenced by genetic factors or other environmental exposures. Additionally, it is unclear whether these associations stem from virus-mediated pathobiology or innate biology that predisposes both poor infection outcomes and asthma. For example, respiratory virus infections may induce either epithelial barrier injury and dysfunction or mucosal immune dysregulation, leading to asthma development.^20^, ^21^, ^22^, ^23^, ^24^, ^25^ Alternatively, children may be born with specific airway or immune defects predisposing them to poor responses to viral infections and the development of asthmatic airway pathobiology.^26^ Answering these questions requires a longitudinal, observational study that characterizes the airway of children from birth through early life, surveils and documents all early-life viral lower respiratory tract illnesses (LRIs), and tracks their progression to asthma diagnosis. Determining which hypothesis is correct carries significant health implications: if viruses indeed induce asthma pathobiology, mitigating LRIs could be an effective strategy for asthma prevention. Supporting this possibility, intervention trials focused on reducing early-life RSV infections have demonstrated efficacy in preventing asthma.^27^ This has led to the development of an RSV vaccine administered during pregnancy and an RSV antibody treatment for infants born during RSV season to block early-life acute RSV infections and inhibit asthma development.^28^^,^^29^

The Puerto Rican Infant Metagenomic and Epidemiologic Study of Respiratory Outcomes, or PRIMERO, birth cohort was established to investigate the prevalence and role of all common respiratory viruses on the development of LRIs and asthma. The GALA I (Genetics of Asthma in Latino Americans) and GALA II (Genes–Environments and Admixture in Latino Americans) studies provided essential insights into asthma risk factors across diverse Latino populations, uncovering genetic, environmental (notably respiratory viruses), and social determinants of asthma susceptibility.^7^^,^^30^, ^31^, ^32^, ^33^, ^34^, ^35^ These studies laid the groundwork for PRIMERO, which extends this research by focusing specifically on the role of respiratory viruses in the development of asthma in Puerto Rican children, a group with one of the highest asthma prevalence rates globally. Specifically, PRIMERO seeks to ascertain whether the association between early-life viral illness and asthma is rooted in deficiencies in the airway at birth or the subsequent alterations of the airway and airway mucosal immune system in response to viral infections.

To achieve this aim, PRIMERO is designed to examine the molecular development and pathobiology of the airway at birth and throughout early childhood, all while performing surveillance for early-life respiratory illnesses (RIs), with viral and molecular response characterization of the detected illnesses. In contrast to prior birth cohort studies, our study utilizes cutting-edge multiplexed molecular assays, which allow us to identify illnesses caused by both RSV and HRV, in addition to metapneumovirus, coronaviruses, parainfluenza, influenza, and other species that have been less studied in this context. Furthermore, by leveraging Puerto Rican’s diverse genetic background with admixture from European, African, and Indigenous American ancestry, PRIMERO children will be genetically characterized to determine if the effects of these viruses on LRI and asthma risk are dependent on genetic variation. This diversity enhances the generalizability of our findings, allowing us to investigate how genetic variation influences asthma risk across a broad ancestral background. Perhaps most innovative, the PRIMERO study will characterize molecular airway dysfunction repeatedly at birth, yearly throughout childhood, and during RIs, through transcriptomic analyses of nasal airway mucosa, collected by swabbing the inferior nasal turbinate. Importantly, our prior work in young children has shown this minimally invasive technique collects both epithelia and airway immune cells, and that the cell types and expression profiles of these upper airway samples mirror those in the lower (bronchial) airways in health and asthma disease.^36^

Leveraging this rich dataset, our goals are 4-fold: (1) identify the genetic and viral determinants of early-life LRIs, (2) elucidate the influence of LRIs caused by different virus species on the development of asthma, (3) delineate the trajectories of airway molecular and cellular development from birth through childhood in both children who do and do not develop asthma, and (4) ascertain whether the airway developmental trajectories leading to asthma are predetermined at birth or influenced by early-life viral LRIs. Beyond these goals, PRIMERO’s unique and comprehensive characterization of RIs and airway pathobiology during childhood offers unprecedented opportunities for further investigation into the impact of prenatal and postnatal social, environmental, and genetic determinants of childhood health and disease.

PRIMERO, a National Heart, Lung, and Blood Institute (NHLBI)-funded prospective observational birth cohort study of Puerto Rican children, is the result of a collaborative effort among research groups from the University of California, San Francisco (UCSF), National Jewish Health (NJH) in Denver, Colorado, and Centro de Neumología Pediátrica (CNP) in Caguas, Puerto Rico. The study meticulously tracks this cohort from birth through early childhood, incorporating RI surveillance during the initial 2 years of life and annual airway sampling during healthy child study visits. Here we provide a comprehensive overview of the objectives, study design, procedures, specimen collections, and recruitment results.

## Methods

### Primary hypotheses

RIs caused by some virus species will independently increase the risk of acute lower respiratory tract symptoms and asthma, while the effect of other species on these outcomes will be dependent on host factors, including genetics. Early-life viral LRIs will directly contribute to the risk of childhood asthma by modulating the early-life development of the airway epithelium and immune system.

### Primary objectives

To address our hypotheses, PRIMERO pursues the following 7 study objectives:

1. Identify the genetic and viral determinants of early-life upper respiratory tract illnesses (URIs) and LRIs and assess the relative impact of different virus species on asthma risk.
2. Determine the association between early-life RIs attributed to various virus species and both the modified asthma predictive index^37^ and asthma disease in childhood.
3. Determine whether the risk of asthma conferred by different viral RIs is dependent on host factors, including genetics.
4. Identify distinct early-life airway gene expression patterns and cellular developmental trajectories, including at early-life milestones and critical transition points, that contribute to asthma in childhood.
5. Determine whether early-life viral LRIs alter airway developmental trajectories.
6. Determine whether the molecular and cellular profiles of the airway at birth are associated with the occurrence of early-life viral LRIs and/or the development of asthma in childhood.
7. Determine whether the gene expression responses to viral LRIs are associated with the subsequent development of asthma.

### Study overview

To address these objectives, we designed PRIMERO as a prospective observational birth cohort study, enrolling newborns and their mothers from March 2020 to June 2023. Surveillance for RIs during the child’s first 2 years involves weekly short message service (SMS) texts and email messages, complemented by in-person visits and biospecimen collections specifically designated for RIs. In addition, newborn and annual follow-up visits for the child’s first 5 years of life are used to assess respiratory health, document environmental and social exposures, measure lung function, and collect biospecimens. The minimum duration of participant follow-up will be 2 years, with the aim to follow participants for a maximum of 10 years pending ongoing support of the study.

### Established community engagement

PRIMERO builds upon our extensive community engagement with the Puerto Rican community, which commenced in 1998 with the initiation of the GALA I study.^30^ This endeavor was designed to explore the heightened rates of asthma prevalence and morbidity among Puerto Rican children. Expanding on this groundwork, our research progressed with the GALA II study spanning from 2008 to 2014.^31^ This case–control study focused on asthma in Latino children, including Puerto Rican participants in our recruitment, epidemiological, and genetic analyses. Our efforts in GALA I and GALA II achieved over an 85% recruitment rate and a follow-up contact rate exceeding 90% among Puerto Rican study participants, with GALA II providing much of the preliminary data for PRIMERO.

Although participants were not directly involved in PRIMERO’s study planning and no formal patient advisory committee was established, PRIMERO benefits from over 25 years of community-centered engagement by the dedicated recruitment team at CNP. Through CNP’s established network with Puerto Rican hospitals and clinics, including the Hospital Interamericano de Medicina Avanzada (HIMA)–San Pablo hospital network, PRIMERO fosters trust and maintains responsiveness to community needs. These partnerships facilitate culturally tailored recruitment and engagement strategies, reflecting the ongoing input and support of the Puerto Rican communities that PRIMERO serves.

### Study population and recruitment

**Fig 1:**
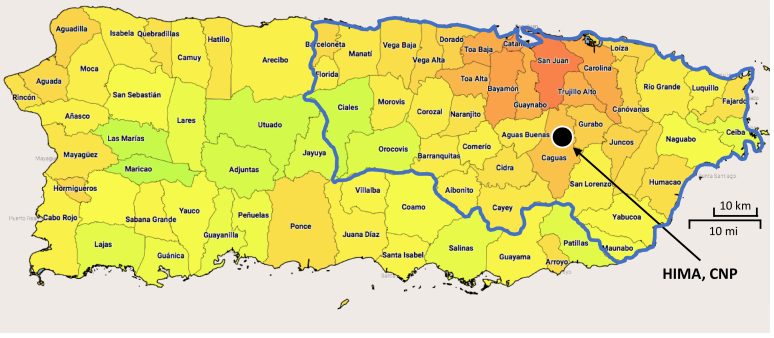
*Redder areas* represent municipalities with higher population densities; *greener areas,* lower population densities. San Juan–Bayamón–Caguas metropolitan area (bordered in *blue*) represents HIMA’s catchment area.

PRIMERO recruited pregnant women who were receiving obstetric/gynecologic (OB/GYN) services and giving birth at HIMA in Caguas, Puerto Rico. Caguas is part of the San Juan–Bayamón–Caguas metropolitan area, which is the most densely populated region in Puerto Rico and includes the capital, San Juan (Fig 1). This metropolitan area houses 63% of Puerto Rico’s population, offering greater access to health care facilities, including specialized services, compared to the island’s more rural areas. Most babies born at HIMA (>95%) come from families residing in this urbanized region. Demographically, the San Juan–Bayamón–Caguas metropolitan area is similar to the entire island in terms of age, marital status, birth rate, and educational attainment.^38^ However, its urban setting provides distinct environmental exposures and health care access that may influence health outcomes compared to less populated, rural regions of Puerto Rico.

Health care providers from HIMA’s OB/GYN services were introduced to PRIMERO via presentations and information sessions. Medical staff were invited to join recruitment efforts, and collaborating offices received PRIMERO brochures for distribution during routine prenatal visits. Women receiving information packets had their details recorded in the custom electronic database, PRIMERO-DB (see Text E1 available in this article’s Online Repository available at www.jaci-global.org). Recruitment occurred in two stages, allowing participants time to consider involvement. Initially, phone calls assessed interest, followed by consent and eligibility determination. PRIMERO recruiters at the HIMA labor and delivery ward used PRIMERO-DB to cross-reference consented participants. The second consent stage occurred after the child was born, before discharge, when mothers were allowed to reaffirm their participation. Protocols and documents were approved by the UCSF institutional review board (no. 420676, reference 18-26263) and a National Institutes of Health–appointed observational study monitoring board, ensuring data safety, study progress, and confidentiality protocols.

### Enrollment, study visits, and procedures

Study visits and procedures are detailed in Table I. Briefly, study participation consists of an enrollment visit, annual visits for the first 5 years of life, and recurring RI visits during the first 2 years of life.

**Table I:** Schedule of study procedures and visits

Visit	Prenatal	Birth	Age 1	Age 2	Age 3	Age 4	Age 5
Recruitment							
Eligibility and informed consent	X	X					
Baseline questionnaire		X					
Cord blood		X					
Maternal blood		X					
Nasal swabs		X					
Annual follow-up visits							
Annual questionnaire			X	X	X	X	X
Anthropometrics			X	X	X	X	X
Nasal swabs			X	X	X	X	X
Blood draw				X			
Impulse oscillometry					X	X	X
Spirometry						X	X
RI surveillance (recurring)							
Weekly SMS text/email messaging		X	X	X			
In-person clinical illness assessments		X	X	X			
RI questionnaire		X	X	X			
URI nasal swabs∗		X	X	X			
LRI nasal swabs†		X	X	X			

∗ First URI per year from birth to age 2.

† All LRI events from birth to age 2.

#### Enrollment

Before birth, expectant mothers in the HIMA OB/GYN clinic expressing interest in the study were assessed for eligibility and consented. Inclusion and exclusion criteria are provided in Table II. At birth, the newborns of consenting mothers were assessed for eligibility, and cord blood was collected. In the recovery ward of HIMA hospital, mothers of eligible infants were approached to supply reconsent. These reconsented mothers were then administered a baseline questionnaire by trained staff, who also collected peripheral blood from the mother and nasal swabs of the inferior turbinates of the newborn. The cord blood collected from infants whose mothers did not reconsent and those infants deemed not eligible was discarded, and these infants were not enrolled onto the study. Blood and nasal samples were processed at CNP, the core facility for our recruitment and follow-up procedures, located in the building adjacent to HIMA. After processing, samples were shipped to each of the laboratory cores: blood to the UCSF Pediatric Asthma Biobank and nasal swabs to the NJH Nasal Airway Biobank (see Text E2 in the Online Repository available at www.jaci-global.org).

**Table II:** PRIMERO enrollment inclusion and exclusion criteria

Subject	Inclusion criteria	Exclusion criteria
Mother	• Residential address in Puerto Rico. • At least 18 years old. • SMS text-enabled phone or working email address. • Can read and understand English or Spanish. • Delivery at HIMA–San Pablo.	—
Newborn∗	• Lives with biological mother. • At least 37 weeks’ gestational age. • Birth weight of at least 2500 g. • Afebrile (<38°C). • No clinical signs of respiratory infection.	• Sibling of a child already enrolled onto PRIMERO. • Multiple birth. • Requires mechanical ventilation. • Immunocompromised. • Congenital or neuromuscular disorders.

To ensure participants are suitable for baseline assessments, some inclusion criteria specify absence of acute symptoms or febrile illness at enrollment. These criteria help maintain the integrity of initial health assessments by reducing potential confounders that could affect respiratory and immunologic baseline measures.

∗ Newborns of at least 36 weeks’ gestation and weighing within 10% of the 2,500 g cutoff (ie, weighing at least 2250 g) at birth were evaluated for inclusion on a case-by-case basis by an obstetrician/pediatrician, who determined by physical examination if the child was otherwise healthy. Only one child of a multiple birth was allowed to enroll. Congenital disorders include pulmonary abnormalities and Down syndrome. Neuromuscular disorders include impaired ability to clear airway secretions.

#### Annual visits and biospecimen collection

Initially, annual visits and questionnaires were aligned with the child’s birthday. However, because of the coronavirus disease 2019 (COVID-19) pandemic, the window was extended to 8 months after the birthday to accommodate stay-at-home mandates and vaccination rollouts. For safety and efficiency, questionnaires are conducted separately by trained PRIMERO staff via phone, utilizing the secure UCSF Research Electronic Data Capture (REDCap) software.^39^^,^^40^ Questionnaires administered at birth and annual follow-up visits during the child’s initial 5 years gather comprehensive data on environmental factors, demographic variables, social aspects, and clinical risk factors associated with recurrent wheezing and asthma. Biospecimens are collected annually during healthy periods, and the modified asthma predictive index is assessed at 2 years.^37^ Biospecimen collection is further described in Text E3 in the Online Repository available at www.jaci-global.org. Annual visits include recording anthropometrics, an eczema assessment, and collection of 2 nasal swabs. In the second year, peripheral blood is processed for allergen sensitization. In the third year, impulse oscillometry is conducted with the plan for spirometry in subsequent years to evaluate lung function. Annual nasal swabs are sent to the NJH Nasal Airway Biobank, and year 2 blood samples go to the UCSF Pediatric Asthma Biobank after processing at CNP.

### RI surveillance

**Fig 2:**
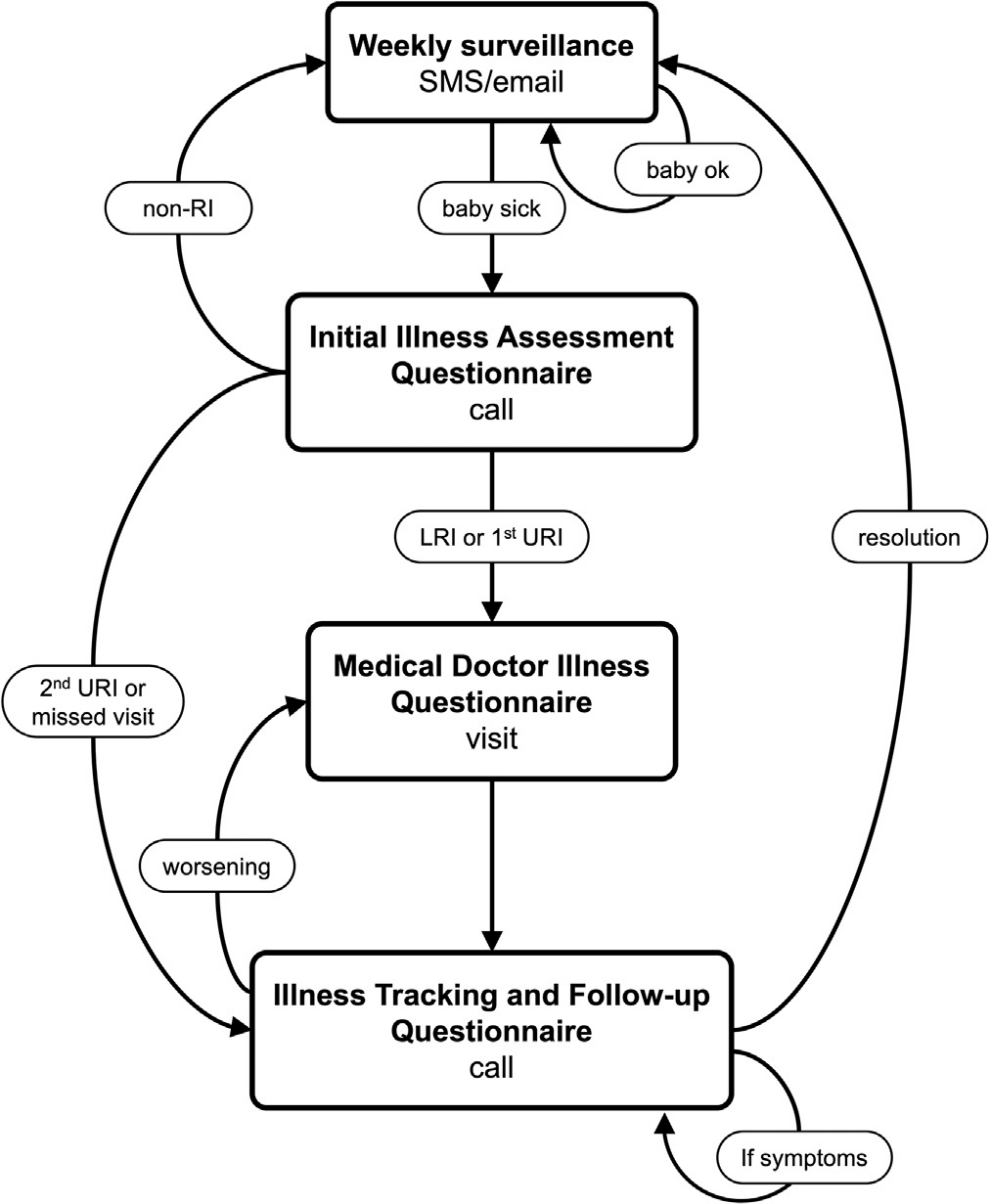
Participants move through statuses assigned at each node to track illness from onset until resolution.

After discharge from the hospital following birth, participants enter the RI surveillance phase of the study (Fig 2), which lasts for the first 2 years of life. Mothers of participating infants receive a weekly automated SMS text or email that provides a hyperlink to indicate whether the child has signs of RI (see Fig E1 in the Online Repository available at www.jaci-global.org). Text and email messages that are unanswered within 24 hours or have an affirmative response are compiled into a weekly call list, followed by a phone call from project staff. In addition to active surveillance, mothers can call PRIMERO staff or visit the clinic for a phone-based or face-to-face assessment. Illness assessments, visits, follow-up, and severity determination are further described in Text E4 in the Online Repository.

### RI outcomes

RI outcomes of the study include the following: identification of early-life LRIs; analysis of RI-associated airway gene expression; determination of respiratory virus infection at a species level; and modified asthma predictive index.^37^ RI outcomes and the research questions we aim to address with these outcomes are described in Text E5 in the Online Repository available at www.jaci-global.org.

### Risk mitigation plan

Study enrollment commenced in March 2020, coinciding with the onset of the COVID-19 pandemic. After considering the risks of pandemic viral exposures to staff and participants, PRIMERO leadership, in conjunction with HIMA, NHLBI, and the observational study monitoring board, decided to proceed with the study, operating under a risk mitigation plan. In addition to enhanced safety measures for PRIMERO participants and staff to limit viral exposure (see Text E6 in the Online Repository available at www.jaci-global.org), anticipated risks included delayed recruitment, participant attrition, and logistical challenges in biospecimen collection and storage. To address these risks, we implemented a range of engagement strategies to enhance participant retention, including regular communication via SMS text/email reminders for study appointments and accessible support for participant questions or concerns. Additionally, we developed a robust biorepository infrastructure to maintain biospecimen integrity. This infrastructure includes standardized protocols for sample collection, transport, and storage, as well as dedicated facilities with controlled temperature settings to ensure long-term preservation and quality control of biospecimens. During the initial pandemic lockdown (March to October 2020), in-person RI assessments were limited to only medically necessary visits for LRIs. However, RI surveillance continued remotely via weekly SMS text and email, enabling data collection on potential URIs, including first URI events in infants. While in-person visits for URIs were temporarily suspended to protect health, this remote tracking permitted insight into URI frequency, although these events lack virus screening. Additionally, in-person visits for COVID-19–positive infants were delayed for 10 days, limiting the proximity of the nasal swab and other biospecimen collections to the acute illness.

### Participant engagement

To enhance retention and engagement, mothers are initially reimbursed for parking costs at HIMA during recruitment, and they receive a thank-you gift at the conclusion of the baseline and each annual follow-up visit. Beyond study visits, participants are regularly contacted through various channels, including SMS texts, email, and the study website (see Fig E2 in the Online Repository available at www.jaci-global.org).

## Results

### Recruitment

**Fig 3:**
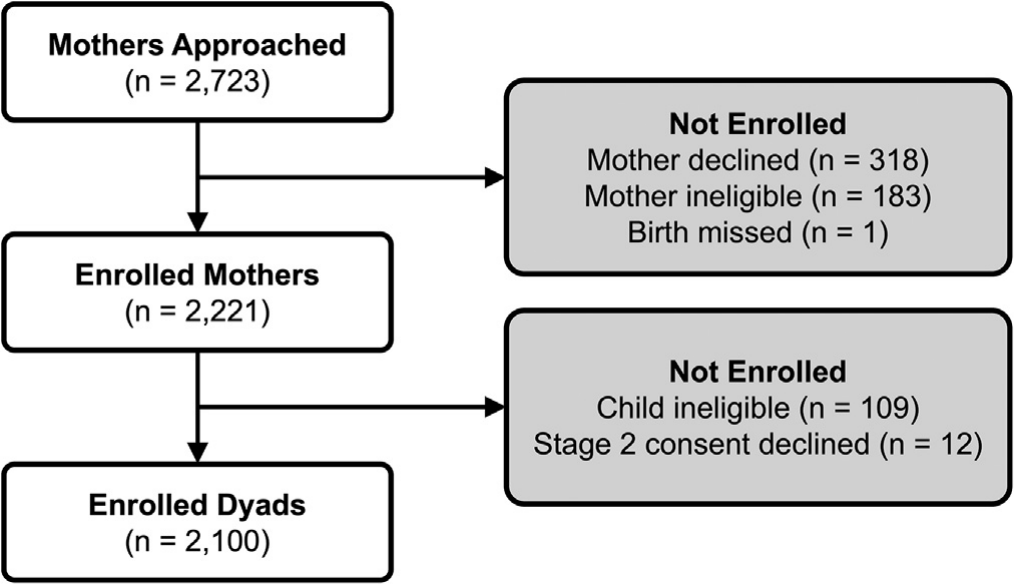
Of 2,723 mothers approached with welcome packets and screened for potential enrollment, 318 mothers declined participation, 183 mothers were excluded as ineligible according to study criteria, and one birth was missed. Additionally, 12 mothers did not reaffirm their consent in study stage 2, and 109 infants were deemed ineligible according to study protocols, resulting in final enrollment of 2,100 mother–child dyads.

Enrollment began in late February 2020, with the first eligible infant born in early March, coinciding with the first COVID-19 cases in Puerto Rico. Despite pandemic restrictions, PRIMERO continued active recruitment until June 2023 (see Fig E3 in the Online Repository available at www.jaci-global.org). During the 40-month period, HIMA delivered 3,543 babies. Mothers delivering on weekends or receiving prenatal care outside HIMA were not considered for recruitment. Among the 2,723 mothers approached, 2,100 mother–child dyads were successfully enrolled (Fig 3), representing 59% of HIMA births.

### Descriptive characteristics of participants

Characteristics of PRIMERO mothers and children (Table III) were gathered from the baseline questionnaire and infant eligibility form. The median (interquartile range) age of mothers was 26.6 (23.1-30.7) years. Cesarean delivery was common (72.6%), with 53.9% unplanned. Children’s race was based on parents’ reported races, with 39.0% White if both parents identified as White, 22.0% Black/African if both parents identified as Black/African, and 39.0% other/mixed if one parent identified as White and the other as Black/African, encompassing all possible combinations.^41^

**Table III:** Characteristics of enrolled mothers and children in PRIMERO

Characteristic	No. (%) or median (IQR)
Maternal age (years)	26.6 (23.1-30.7)
Cesarean deliveries	1,524 (72.6)
Unplanned Cesarean	821 (39.1)
Gestational age (weeks)	38.7 (38.0-39.4)
Birth weight (g)	3,202 (2,915-3,466)
Child’s sex (male)	1,048 (49.9)
Child’s race∗	
Black/African	461 (22.0)
More than one race	816 (38.9)
White	820 (39.0)
Some other race	2 (0)
Perinatal antibiotics exposure†	433 (20.6)
Maternal prenatal smoking‡	22 (1.0)
Maternal education status, low§	739 (35.2)
Paternal education status, low§	946 (45.0)

*IQR,* Interquartile range.

∗ Child’s race was derived from parents’ reported races: “White” if both parents identified as White, “Black/African” if both parents identified as Black/African, and “other/mixed” if one parent identified as White and the other as Black/African.

† Perinatal antibiotic exposure if neonate was administered antibiotics in last trimester or neonate was administered antibiotics soon after birth.

‡ Maternal prenatal smoking if mother reported smoking during pregnancy or reported time from quitting is less than child’s gestational age.

§ Low educational status if reported highest level of education as “less than high school education” or “high school education or GED.”

### Participant retention

As of April 29, 2024, 2,069 study participants remain actively engaged, while 148 are on standby status after requesting reduced contact frequency, relocating from Puerto Rico, or being unable to commit to future follow-up. Additionally, 36 participants withdrew from the study, and 1 child died of reasons unrelated to the study.

### Annual visits and biospecimen collection

Among children eligible for annual visits (Table IV), 60% of expected visits occurred and 71% of expected questionnaires were completed as of April 29, 2024. Nasal swabs were successfully collected from 90% of these children, and blood samples were obtained from 97%. Among children 3 years and older who attended their third annual visit, 51% have completed impulse oscillometry.

**Table IV:** Annual assessments during first 2 years of life in PRIMERO children as of April 29, 2024

Outcome	Year 1	Year 2∗
Total expected visits, assessments, and specimens	2,014	1,373
Completed questionnaire (% of expected)	1,742 (86%)	993 (72%)
Completed visits (% of expected)	1,352 (67%)	840 (61%)
Completed nasal swabs, passed QC (% of expected annual visits)	1,252 (93%)	770 (92%)
Completed CBC processed, passed QC (% of expected, year 2 visits)	—	817 (97%)
Completed allergen testing, passed QC (% of expected, year 2 visits)	—	747 (89%)

*CBC,* Complete blood count; *QC,* quality control.

∗ Percentages of year 2 completed questionnaires and visits exclude the 10% and 14% of subjects, respectively, who remained eligible but had not yet completed them.

After an initial decline in annual follow-up visit rates, multiple engagement strategies were implemented to encourage participants to attend their yearly visits. These included regular updates to web-based study materials featuring study milestones and sending personalized birthday and holiday greetings from the study team. For participants without web access, engagement materials were printed for display in our observation room. Additionally, reminder posters were placed in clinic corridors, highlighting benefits like free allergen testing for children nearing their second birthday. Furthermore, a newly developed REDCap tool now aids schedulers in efficiently identifying participants due for annual questionnaires and visits, helping ensure assessments are completed on time. Finally, outreach hours were extended to evenings, allowing caregivers with daytime commitments more flexibility.

### RIs

Through April 29, 2024, RI surveillance identified a total of 6,076 illnesses, of which 61.6% (n = 3,744) and 38.4% (n = 2,332) were URIs and LRIs, respectively (Table V). Of the LRIs, 60.9% (n = 1,421) were mild/moderate, and 39.1% (n = 911) were severe.

**Table V:** RIs monitored during the first 2 years of life in PRIMERO children as of April 29, 2024

Outcome	Participants with ≥1 illness, no. (% of cohort)	Illness count, no. (% of RIs)	Illness count per participant with ≥1, median (IQR)	Age (months) at time of first illness, median (IQR)
URI	1,402 (67.8%)	3,744 (61.6%)	2 (1-4)	6.7 (3.5-11.6)∗
Mild/moderate LRI	828 (40.0%)	1,421 (23.4%)	1 (1-2)	9.6 (5.0-15.1)†
Severe LRI	538 (26.0%)	911 (15.0%)	1 (1-2)	10.2 (6.6-15.8)

*IQR,* Interquartile range.

∗ Age in months at time of first illness for participants with at least one URI, but no LRIs.

† At least one mild/moderate LRI, but no severe LRIs.

Throughout the COVID-19 pandemic, in-clinic assessments for medically necessary LRI events continued, with approximately 70% of all LRI events receiving in-person evaluations and a high compliance rate for nasal swab collection (93% of in-person cases). This approach upheld protocol adherence and ensured data continuity for critical RI events despite pandemic-related restrictions. After the lifting of the face mask mandate and vaccine requirements for employees and public entry in March 2022, the in-person evaluation rate for LRI events rose to approximately 83%, reflecting improved adherence to the study protocol and enhancing the consistency and comprehensiveness of data collection.

### Adverse events

Related adverse events are defined as any unfavorable medical occurrence attributed to a PRIMERO procedure, such as blood draw, nasal swab, or pulmonary function testing. A singular adverse event was recorded, involving a syncopal episode during a child’s blood draw.

## Discussion

PRIMERO was designed to characterize the relationship between viral RIs in early life and the subsequent development of childhood asthma, focusing on the pivotal role of airway epithelial dysfunction. PRIMERO sets out to determine whether this dysfunction manifests at birth or emerges as a consequence of viral RIs during infancy and early childhood. Our prospective birth cohort study is poised to yield groundbreaking insights into the genetic and viral risk factors associated with severe RIs. Furthermore, it aims to pinpoint distinct airway cellular developmental trajectories observable in children at high risk for asthma at birth, after RIs, and during periods free of respiratory symptoms preceding the onset of asthma. Furthermore, the wealth of additional data collected on PRIMERO participants will allow other investigations into the early life social, environmental, and genetic determinants of severe reparatory wheezing illnesses and subsequent asthma.

*Primero* means “first” in Spanish, signifying PRIMERO as the inaugural asthma birth cohort study in Puerto Rico. The imperative for comprehensive research across diverse genomes cannot be overstated, as it is fundamental to advancing science and medicine.^42^^,^^43^ Despite significant attention and resources directed toward genetic research, globally diverse populations remain notably underrepresented.^44^ Here, PRIMERO is helping to bridge this gap toward equal representation of study populations in biomedical science. The emphasis of PRIMERO on the population of Puerto Rico is emblematic of the NHLBI’s strategic vision, which underscores the exploration of factors contributing to health disparities among diverse populations.^45^ Additionally, recruitment and follow-up activities are exclusively conducted within Puerto Rico, in close partnership with local investigators, clinicians, and other Puerto Rican professionals, enriching the biomedical workforce with diversity and expertise.

A fundamental strength of PRIMERO lies in its innovative approach, capitalizing on the minimally invasive nature of nasal airway swabs to procure critical respiratory samples at multiple times: at birth, during early-life RIs, and annually for the first 5 years of life.^32^^,^^36^^,^^46^ Our study utilizes cutting-edge multiplexed molecular assays, enabling the identification of a spectrum of RI-causing viruses, including RSV, HRV, metapneumovirus, coronaviruses, parainfluenza, influenza, and other less studied species. Collected specimens from PRIMERO participants undergo genetic characterization, allowing us to determine the role of genetic variation in the effects of these viruses on LRI and asthma. Most notably, through transcriptomic analyses of nasal airway mucosa, the PRIMERO study introduces a groundbreaking approach by longitudinally characterizing molecular airway dysfunction at birth, annually throughout childhood, and during RIs in the first 2 years of life. Future studies could leverage the biorepository of samples collected at multiple time points for other multiomics analyses, including epigenetic assessments. Such analyses could provide insights into how early-life respiratory infections and other exposures contribute to asthma risk through epigenetic modifications, offering an additional layer of understanding in RI outcomes and asthma pathogenesis. Extensive research substantiates the effectiveness and reliability of nasal swabbing, underscoring critical findings: it reveals that the upper and lower airways share similar cell types and expression patterns, and upper airway insults can elicit lower airway symptoms and immune responses.^36^^,^^47^, ^48^, ^49^, ^50^ The inflammatory endotypes of asthma observed in both the nasal and bronchial airways exhibit a high degree of correlation, emphasizing the importance of the nasal airway epithelium as a primary site for infection with asthma-associated respiratory viruses.^36^ Furthermore, we previously demonstrated the versatility of RNA-sequence data generated from nasal airway epithelial swabs, serving a dual purpose: elucidating host transcriptional profiles and screening samples for pathogenic respiratory viruses through metagenomic analysis.^34^ This single-swab RNA-sequence approach offers a robust and tightly controlled assessment of the host response to viral infection and stands as a central analysis method within our study.

As with any complex study, PRIMERO has its unique challenges. One challenge was the low birth rate in Puerto Rico, which, at 0.90 births per woman, is approximately half that in the mainland United States.^51^ Despite the relatively small source population of potential participants, PRIMERO recruitment has been overwhelmingly successful. Another challenge was the lack of available software to manage the recruitment, surveillance, and follow-up of 2,100 mother–child dyads and to interact with their electronic medical record as they progress through the study. A key innovation was the creation of PRIMERO-DB to address this need. PRIMERO-DB has the same Health Insurance Portability and Accountability Act securities as electronic medical record data and is available from any web-enabled device, providing user-specific permissions to authorized users. Finally, PRIMERO recruitment began just as the COVID-19 pandemic unfolded, introducing unique challenges. Pandemic control measures, such as limited indoor capacity in OB/GYN offices, complicated traditional rapport-building through face-to-face interactions. Our data collection was also affected: in-person nasal swab collections and transcriptomic analyses for COVID-19–positive infants were performed after a 10-day waiting period, and presumptive URIs were monitored remotely during the initial lockdown (March to October 2020). Although remote tracking allowed us to document all illnesses, it limited our ability to identify and analyze viral agents in some URI cases. Given these constraints, assessing the generalizability of our findings to infants born both before and after the pandemic is crucial. Moreover, PRIMERO is uniquely positioned to investigate how major lockdown and reopening phases in Puerto Rico, with their accompanying periods of strict social distancing, influenced RIs, immune development, and asthma risk in early childhood.

Creating a birth cohort uniquely positions us to answer questions about the early-life origins of health and disease. We aim to leverage this rare opportunity to study the broader health of children over the next decades. Given adequate support, long-term prospective follow-up of PRIMERO participants will enable studies of numerous exposures and outcomes and enable the identification of biomarkers that may predict outcomes well before their traditional diagnosis. This information collectively promises insights into potential pathways that may be targetable in efforts to prevent illnesses and the subsequent development of childhood diseases such as asthma.

**Clinical implications**

PRIMERO, Puerto Rico’s first asthma birth cohort study, is poised to address long-standing questions about childhood asthma development, with the potential to benefit children of all racial and ethnic backgrounds.

## Disclosure statement

Supported in part by the Sandler Family Foundation; the American Asthma Foundation; the Amos Medical Faculty Development Program from the Robert Wood Johnson Foundation; the Harry Wm. and Diana V. Hind Distinguished Professor in Pharmaceutical Sciences II; the American Lung Association (CAALA2023); the Wohlberg and Lambert Endowed Chair of Pharmacogenomics (M.A.S.); and the National Institutes of Health/National Heart, Lung, and Blood Institute (5U01HL138626 and 1K23HL169911). The content of this publication is solely the responsibility of the authors and does not necessarily reflect the views or policies of the US Department of Health and Human Services, nor does mention of trade names, commercial products, or organizations imply endorsement by the US government.

Declaration of generative AI and AI-assisted technologies in the writing process: During the preparation of this work, the authors used ChatGPT 4o, Perplexity, and Grammarly to improve language and readability. After using these tools/services, the authors reviewed and edited the content as needed. They take full responsibility for the content of the publication.

Disclosure of potential conflict of interest: S. Oh is currently employed by Amgen Inc. M. A. Seibold reports receipt of research funding from Genentech, Medimmune, and Pfizer. D. Sheppard is a scientific founder of Pliant Therapeutics. The rest of the authors declare that they have no relevant conflicts of interest.
