# Supplementary material for: The PRIMERO birth cohort: Design and baseline characteristics
Source: J Allergy Clin Immunol Glob. 2025 Apr 11;4(3):100470. doi: 10.1016/j.jacig.2025.100470 (PMC12140944; doi:10.1016/j.jacig.2025.100470)
Supplement: Supplementary Figure 1 [file mmc1.docx]

**Supplemental Figure 1.** Example SMS text received (left panel) and linked page (right panel) by which PRIMERO participants respond to weekly message enquiring about their child’s health.

| **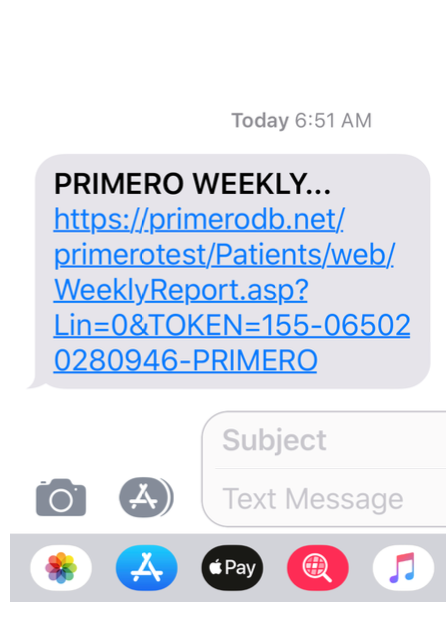** | **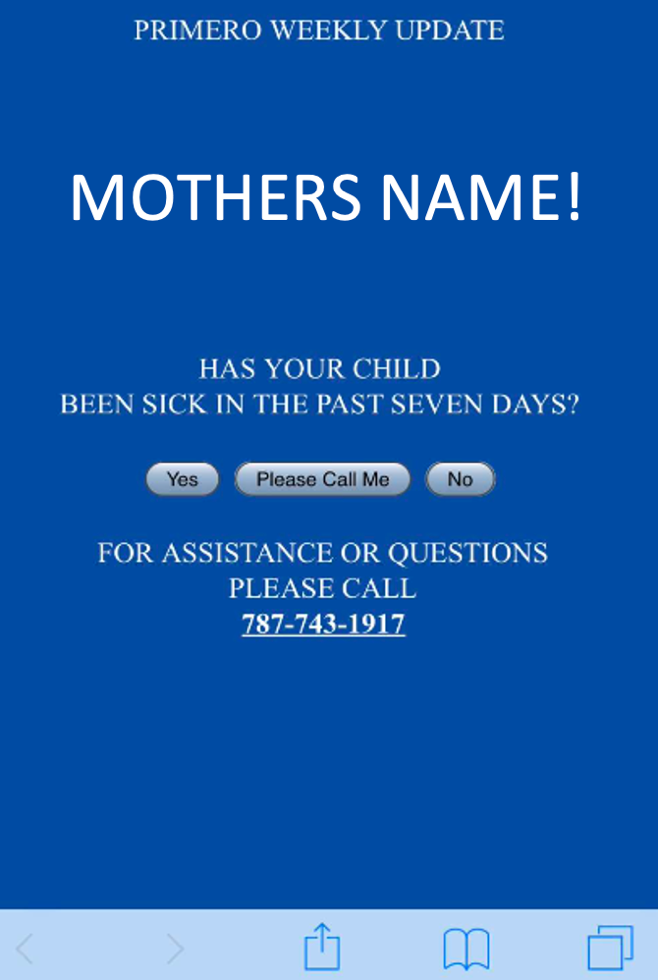** |
| --- | --- |
