# Supplementary material for: The PRIMERO birth cohort: Design and baseline characteristics
Source: J Allergy Clin Immunol Glob. 2025 Apr 11;4(3):100470. doi: 10.1016/j.jacig.2025.100470 (PMC12140944; doi:10.1016/j.jacig.2025.100470)
Supplement: Supplementary Figure 2 [file mmc2.docx]

**Supplemental Figure 2.** Example participant engagement through study website updates, and personalized greetings.

**
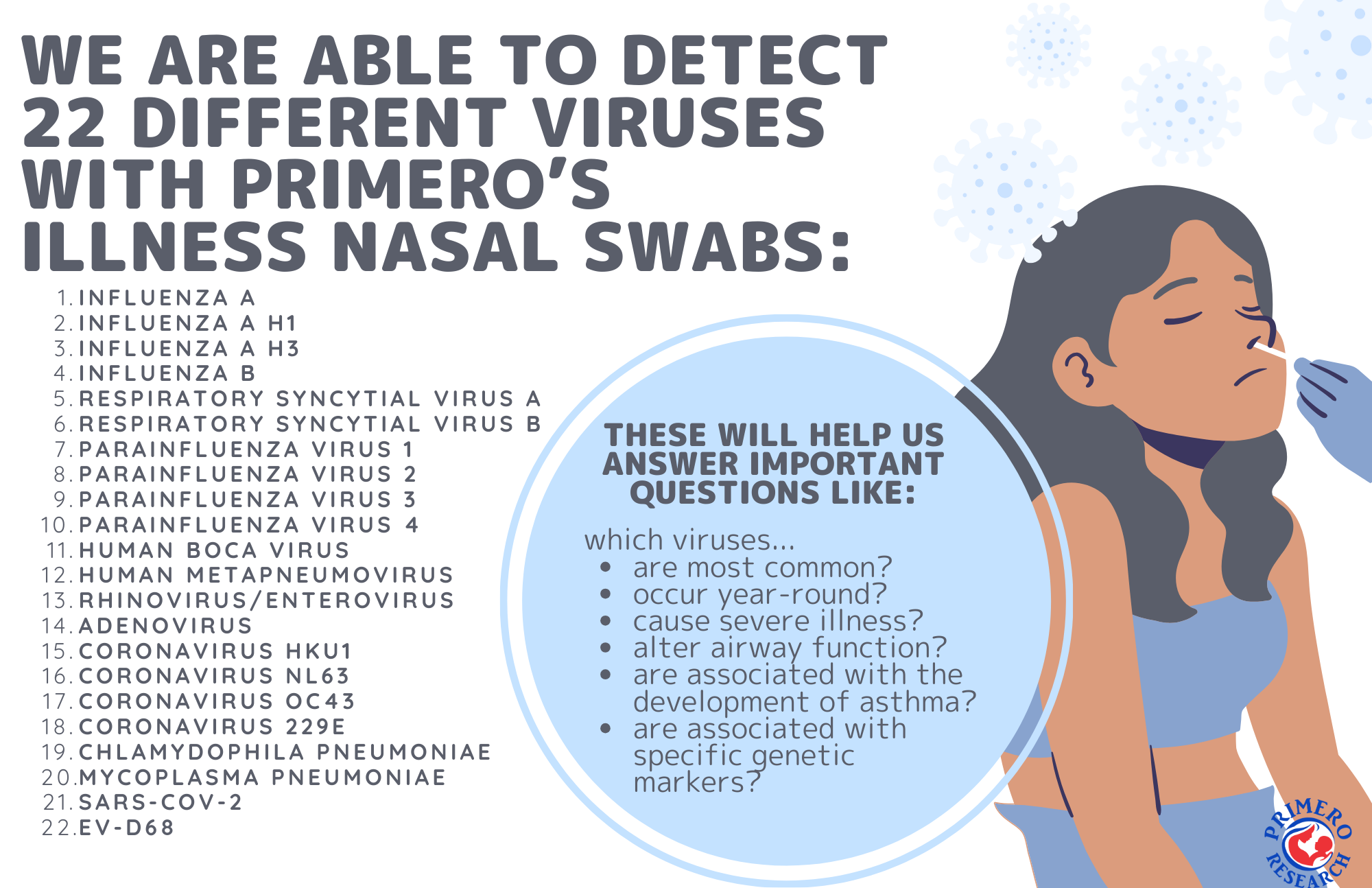
**

The study website and newsletters serve to keep participants informed about the study's progress and provide prompts for updating contact information. Weekly contacts during the first two years help identify RIs, and personalized SMS texts with a link to the study website are sent on the child participants’ birthdays. Additionally, participants receive annual holiday greetings via email. All engagement materials are available and distributed in the participant’s preferred language.
