# Supplementary material for: The PRIMERO birth cohort: Design and baseline characteristics
Source: J Allergy Clin Immunol Glob. 2025 Apr 11;4(3):100470. doi: 10.1016/j.jacig.2025.100470 (PMC12140944; doi:10.1016/j.jacig.2025.100470)
Supplement: Supplementary Figure 3 [file mmc3.docx]

**Supplemental Figure 3.** Monthly and cumulative PRIMERO enrollment from March 2020 to June 2023


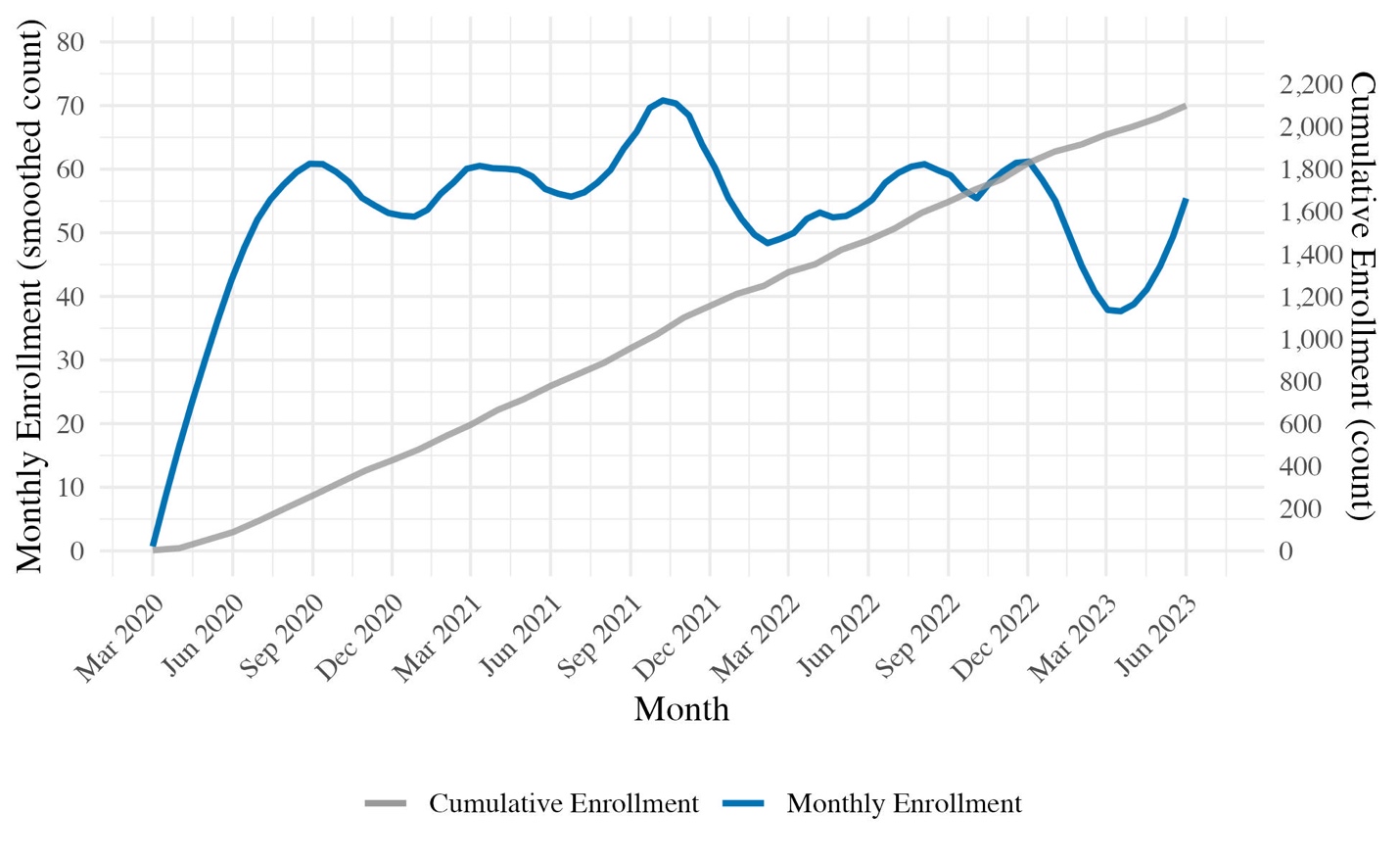


The inaugural eligible infant entered the study in March 2020, and a median of 55 infants were enrolled each month thereafter. The PRIMERO cohort consists of 2,100 term healthy infants, with enrollment concluding in June 2023. Smoothed count: the Locally Weighted Scatterplot Smoothing (LOESS) method was used to fit a smooth curve through the monthly enrollment count data points.
