# Supplementary material for: The PRIMERO birth cohort: Design and baseline characteristics
Source: J Allergy Clin Immunol Glob. 2025 Apr 11;4(3):100470. doi: 10.1016/j.jacig.2025.100470 (PMC12140944; doi:10.1016/j.jacig.2025.100470)
Supplement: Supplementary Material [file mmc4.docx]

**SUPPLEMENTARY TEXT**

**Supplemental Text 1: Data Collection, Management, and Quality Assurance**

PRIMERO-DB

Given the scale and complexity of the study (e.g., coordinating recruitment visits, study visits, and the unique procedures associated with each visit), the team developed an electronic record-keeping software *de novo* to track these tasks for all participants as they progress through each stage of the study. The system, PRIMERO-DB, is used to manage recruitment, surveillance, and follow-up activities, and is implemented with the same Health Insurance Portability and Accountability Act (HIPAA) securities as electronic medical record (EMR) data. Potential participants were first entered into PRIMERO-DB when recruiters informed pregnant women about the study. Women interested in the study were asked to offer their name, phone number, and expected due date (stage 1 consent must have occurred prior to the child’s birth). This information was entered into a tracking log in PRIMERO-DB, which in turn helped recruiters to prioritize informed consent and eligibility determination on a daily basis by providing a list of women sorted by due date. Upon presentation of the mothers to labor and delivery, PRIMERO-DB was used to identify consented mothers and manage the flow of tasks. Admitted women were cross-referenced against the PRIMERO-DB tracking log to identify consented and eligible mothers. Once the child was born, PRIMERO staff recorded the collection of cord blood from consented participants by scanning barcoded collection tubes into PRIMERO-DB. This in turn notified laboratory technicians to prepare for the arrival of samples and to scan them as received once they arrived at the laboratory. Reaffirmation of consent, determination of infant eligibility, and administration of the baseline questionnaire were also conducted through PRIMERO-DB. Automated weekly text/email messages asking participants about their child’s respiratory health, and their responses (or lack of) are sent through, and automatically recorded in PRIMERO-DB once the mother and child leave the hospital and enter the illness surveillance phase of the PRIMERO study.

UCSF REDCap

The UCSF Research Electronic Data Capture (REDCap) system is used to collect, transfer, store, and manage recruitment and annual follow-up visit data. REDCap is a professionally managed, secure, HIPAA-compliant, web-based system for building and managing web-based research.

Data Quality Assurance

The UCSF Data Coordinating Center (DCC) monitors study protocol compliance and ensures data quality through range and logic checks at time of data entry, and through quality control assessments of the study database on a regular basis. PRIMERO staff are assessed for data completeness, data entry error rates, and internal consistency of collected data. Staff receive regular feedback on these metrics and opportunities for continued training on study protocols as necessary.

**Supplemental Text 2: Core Facilities and Sample Handling**

PRIMERO activities are coordinated through three core facilities:

Recruitment and Follow-up Core

CNP is the recruitment and follow-up core and coordinates activities between recruiters, data collectors, and staff that perform follow-up activities. CNP houses the text messaging and call center for RI surveillance and study visit scheduling. For PRIMERO, CNP constructed a negative-pressure biosafety level 2 laboratory with the capabilities of [1] processing, culturing, and storing nasal swab specimens, and [2] processing and isolating buffy coat and plasma samples for cryopreservation from maternal and infant cord and peripheral blood samples. Additionally, in response to the COVID-19 pandemic, the CNP constructed a negative-pressure clinical examination room for patient visits.

Data Coordination Core

The UCSF Asthma Collaboratory is the data coordinating center (DCC) and the IRB of Record for all sites. The DCC helps collect, monitor, integrate, and distribute information for PRIMERO. The DCC generates and distributes data collection forms and protocols and maintains a coded data set on UCSF servers.

UCSF Laboratory Core

Biological specimens from the child are obtained from cord blood (cord blood mononuclear cells [CBMCs], plasma, deoxyribonucleic acid [DNA], and ribonucleic acid [RNA]) and during the year 2 annual visit (serum, plasma, DNA, and RNA); biological specimens from the mother (plasma, DNA, and RNA) are collected 24 hours postpartum. CBMCs and plasma extractions are performed at CNP. All biological specimens are coded and shipped to the UCSF Pediatric Asthma Specimen Bank (IRB# 10-00085) on dry ice. UCSF utilizes a Microsoft Access database to manage all biological specimens. Blood tubes are further processed at UCSF for DNA utilizing the Wizard® Genomic DNA Purification Kit (Promega, Fitchburg, WI).

NJH Airway Biobank Core

Two coded nasal airway swab samples are collected from each visit at CNP for [1] nucleic acid isolation, and [2] cryopreservation. Following collection in appropriate buffers optimized for downstream analyses, airway specimens are shipped to the NJH Airway Biobank Core in Denver, Colorado. For nucleic acid isolations, NJH uses a modified DNA/RNA isolation protocol using a Beckman Coulter Automated i7 Liquid Handling Workstation fitted with the Data Acquisition and Reporting Tool (DART) software for sample tracking and extraction of both DNA and RNA biomolecules prior to banking. Swabs collected for cryopreservation are banked in liquid nitrogen for downstream culture, *in vitro* experiments, and single-cell RNA sequencing (scRNA-seq) outcomes and analyses. CNP and NJH utilize both FreezerWorks and LabGuru electronic laboratory management systems to maintain sample tracking of shipments between sites and for extraction and inventory management of all PRIMERO biospecimens.

Addressing Operational Challenges for a Remote Study Site

For investigators based on the U.S. mainland, operating a study site removed from the continental U.S. can amplify the challenges of conducting a research study. We leveraged our experience working in Puerto Rico since the late 1990s to obviate some of these challenges. For example, blood samples from an individual are divided into two separate shipments and the second shipment is not sent until the first shipment has been received. In the event that a shipment is delayed while in transit and samples are lost because they were not maintained at the proper temperature (e.g., ice packs have fully melted), the second batch will not be sent until the reasons for the delay have been addressed. Past solutions have included switching couriers, adding more ice packs, and re-training staff. We also keep abreast of current events, including civil disturbances and weather phenomena. We have installed a -80°C freezer and self-sustaining liquid nitrogen storage capabilities at CNP to delay shipment of study samples if we anticipate that significant events may have the potential to affect a proper delivery. Laboratory information management system software is used to track the location and status of all study samples in real time.

Procurement Strategies

Procurement of study materials (nasal swabs, blood tubes, personal protective equipment, etc.), had been challenging with pandemic-related interruptions to supply chains. Thus, we have been diligent in securing new and back-up vendors, and in the stockpiling of enough supplies to allow study operations for the PRIMERO study to continue for at least two months.

**Supplemental Text 3: Biospecimen Collection**

Cord and maternal blood are collected at enrollment to define genome-wide genetic variation in mothers and children. Nasal swabs are collected from newborns at enrollment, at each annual visit, and during in-person RI visits. Illness swabs are tested for SARS-CoV-2 and enterovirus D68 using PCR assays and for 22 other common respiratory pathogens using the NxTAG Respiratory Pathogen Panel. Nasal swabs are also used to evaluate longitudinal airway gene expression in health and illness using whole transcriptome RNA-sequencing. In addition, blood is collected at the year 2 visit to obtain complete blood counts with differentials and assess allergen sensitization via specific IgE testing to perennial aeroallergens and foods. In addition to calculating the mAPI, these laboratory assessments will yield peripheral eosinophil counts and early allergen sensitization profiles, which may interact with viral exposures to influence asthma risk.

**Supplemental Text 4: Respiratory Illness Surveillance**

Initial Illness Assessment

Mothers who report signs of illness in their child are contacted by project staff via phone. Project staff administer an Initial Illness Assessment Questionnaire (IIAQ), a high-sensitivity, low-specificity screening questionnaire designed to capture all LRIs based on the presence of symptoms and/or having seen a healthcare provider because of the illness. All likely LRIs are referred for an in-person clinic visit. The PRIMERO study protocol planned for one in-clinic URI assessment per child per year. URI visits were briefly paused under Puerto Rico’s COVID-19 stay-at-home mandate from March 15, 2020 to September 30, 2020, resuming after mandates were lifted.

Respiratory Illness Visits

All LRI events are invited for an in-clinic visit and nasal swab collection, whereas only one URI event per child per year is assessed in-clinic. This approach balances the stronger association between LRIs and asthma risk with logistical considerations, as the high frequency of URIs would make frequent clinic visits impractical for families. During in-person visits, a clinical evaluation including a Medical Doctor Illness Questionnaire (MDIQ) and nasal swab are completed. The Pediatric Respiratory Assessment Measure (PRAM) and the Respiratory Severity Score (RSS) are administered to obtain a clinically standardized measure of the child’s illness severity. A determination of URI versus LRI is recorded on the MDIQ based on the physician’s assessment, PRAM, and RSS scores. A final determination of upper versus lower respiratory tract illness will be recorded on the MDIQ based on the physician’s assessment of the child’s current and retrospective reported symptoms. Specifically, any of the following will result in the illness being classified as an LRI:

- Clinician assessed wheeze
- Accessory muscle use
- O2 saturation < 92%
- PRAM score > 3 (a score of 3 can be obtained by air entry criteria, which can be subjective and an O2 saturation of 92-94%)
- RSS score of ≥ 4 (denotes a greater than 80% probability of LRI)
- Self-report of symptoms consistent with LRI (i.e. wheeze, cough that interfered with daily activities, cough that interfered with sleep, fast breathing or gasping for air).

The choice of these cut points for the PRAM and RSS assure that all individuals with a higher probability of LRI based on these scores will be coded as LRI. The PRIMERO physician will collect nasal swabs from the child at the conclusion of the respiratory illness surveillance visit.

Illness Follow Up

All RIs are tracked using the Illness Tracking and Follow-up Questionnaire (ITFQ) administered by phone following a RI visit or IIAQ without a scheduled face-to-face visit. PRIMERO staff conduct weekly ITFQs to document the trajectory of the RI and to help determine the current state of the illness, presence and severity of symptoms, and occurrence of various illness-related clinical events (e.g., diagnosis of bronchiolitis; prescription of oral steroids; illness-related hospitalizations). Once a RI is determined to have resolved, the child is returned to regular weekly SMS-text/email surveillance messaging.

Determination of Illness Type and Severity

As not all illnesses are assessed in-clinic, illnesses are classified into LRIs and URIs based on symptom report. LRIs report one or more of the following symptoms: a cough that interferes with daily activities, the presence of wheezing/whistling in the chest, fast breathing or gasping for air, and sleep disturbed by cough, wheeze, or difficulty breathing. Participants reporting only to have a runny/plugged nose, sneezing, and/or a mild cough are considered most likely to have a URI. Participants not reporting any respiratory symptoms are considered to have a non-respiratory tract illness (non-RI) and are advised to continue to monitor their child and seek care with a healthcare provider should their child’s illness worsen. LRIs are further dichotomized as mild/moderate or severe. LRIs are classified as severe if the illness requires hospitalization or prescription of oral steroids.

**Supplemental Text 5: Respiratory Illness Outcomes and Associated Research Questions**

Identification of Children with Early-life LRIs

Participants will be classified at the end of their 2-year surveillance period based on the severity of LRI(s) experienced: did not experience an LRI; experienced at least one mild/moderate LRI, but not a severe LRI; or experienced at least one severe LRI.

Analysis of Respiratory Illness-Associated Airway Gene Expression

We will use RNA sequencing to measure whole transcriptome nasal airway epithelium gene expression patterns at birth, during defined URI and LRI events, and from annual follow-up visits from ages one through five.

Due to COVID-19 lockdowns, PRIMERO relied on SMS/email surveillance to gather data on URI occurrences from March to October 2020, capturing presumed URI events without in-person assessment or biospecimen collection. Additionally, in-person visits for COVID-positive infants were delayed for 10 days, limiting the proximity of the nasal swab and other biospecimen collections to the acute illness. Despite these restrictions on visits for known COVID cases, we have already identified 39 URIs and 37 LRIs assessed in person with a SARS-CoV-2 positive nasal swab. Therefore, gene expression responses to SARS-CoV-2 infections can still be evaluated in both URIs and LRIs.

Determination of Respiratory Virus Infection at a Species Level

Viral species-specific data generated from nasal airway swab RNA and metagenomic analysis of RNA-sequence data will allow us to determine if a participant has been infected with a respiratory virus and determine if viral species are associated with illness severity outcomes.

mAPI

Children undergo clinical assessment for asthma risk at two years of age via the mAPI, which is an updated iteration of the asthma predictive index (API) and provides an assessment for future asthma risk (51). The mAPI has greater sensitivity than the API and has been included in the National Asthma Education and Prevention Program (NAEPP) Expert Panel Report 3 (EPR-3) guidelines as a criterion for the institution of long-term therapy to decrease asthma morbidity and exacerbations (52).

Secondary Outcomes

Since we will have viral qPCR results from all documented LRIs for common respiratory virus species (HRV-A, HRV-B, HRV-C, RSV, metapneumovirus, parainfluenza, influenza), and previous findings suggest that the specific virus responsible for the infection may influence asthma risk, we will examine the following virus-informed secondary outcomes:

1. Early-life viral RI outcome. We will use viral qPCR results from all documented LRIs for common respiratory virus species (HRV-A, HRV-B, HRV-C, RSV, metapneumovirus, parainfluenza, influenza) to reclassify the early-life RI outcome by confirmed viral LRIs. Specifically, new groups will be defined as follows:
   - Group 1: no viral LRIs;
   - Group 2: at least one mild/moderate viral LRI but no severe viral LRIs;
   - Group 3: at least one severe viral LRI.
2. Early-life HRV or RSV RI outcome. The relationship between early-life viral LRIs and asthma is strongest for RSV and HRV. Therefore, we will reclassify the early-life RI outcome as follows:
   - Group 1: no HRV or RSV LRIs;
   - Group 2: at least one mild/moderate HRV or RSV LRI but no severe HRV or RSV LRIs;
   - Group 3: at least one severe HRV or RSV LRI.
3. Early-life HRV RI outcome. To identify specific effects of HRV, we will reclassify the early-life RI outcomes as follows:
   - Group 1: no HRV LRIs;
   - Group 2: at least one mild/moderate HRV LRI but no severe HRV LRIs;
   - Group 3: at least one severe HRV LRI.
4. Early-life RSV RI outcome. To identify specific effects of RSV, we will reclassify the early-life RI outcomes as follows:
   - Group 1: no RSV LRIs;
   - Group 2: at least one mild/moderate RSV LRI but no severe RSV LRIs;
   - Group 3: at least one severe RSV LRI.

We will perform six secondary analyses designed to answer the following research questions involving these 4 alternate, viral-informed reclassifications of the early-life RI outcomes.

1. What are the genetic determinants of airway gene expression at birth, in illness, and at two years of age?
2. What is the prospective association of newborn airway gene expression with early-life RI outcomes?
3. What is the association of year two airway gene expression with early-life RI outcomes?
4. What changes in airway gene expression from birth to two years of age are a consequence of early-life RI outcomes?
5. What is the association of early-life RI outcomes with asymptomatic virus infection at two years of age?
6. What is the association of early-life RI outcomes with the modified asthma predictive index (mAPI) outcome at two years of age?

Genetic ancestry analysis will be used to capture the genetic heterogeneity within the PRIMERO cohort. Global ancestry scores, calculated from genome-wide data, allow for adjustment of population structure in analyses, ensuring that associations accurately reflect the genetic complexity of the study population.

We will perform sensitivity analyses to assess the potential effects of COVID-19 lockdowns on study outcomes. For example, we will evaluate outcomes in analyses stratifying participants by recruitment timing relative to different COVID-19 lockdown periods. This stratification will allow us to assess whether variation in early-life exposures resulting from pandemic restrictions, such as reduced exposure to respiratory pathogens and altered social interactions, has an effect on our results. In particular, we will assess whether the lockdowns impacted early-life illness and asthma outcomes, as well as airway developmental trajectories.

Patterns and determinants of missing data for key study modules, including missed visits and assessments, will be systematically evaluated. Determinants such as socioeconomic status, educational attainment, race, and birth characteristics will be examined for associations with missing data. Weighted analyses may be used to account for potential biases, ensuring robust and generalizable findings.

Finally, the study’s inclusion of comprehensive family health histories and mother-child dyad genome-wide genotyping allows for unique insights into transgenerational influences on respiratory and immune health. Future research building on PRIMERO’s design may also consider epigenetic assessments to further explore these intergenerational effects.

**Supplemental Text 6: COVID-19 Precautions (Risk Mitigation Plan)**

The risk mitigation plan initiated enhanced safety measures for PRIMERO staff when interacting with participants and limited face-to-face interactions to medically necessary visits. Specifically, study activities related to birth and LRI visits were allowed to proceed since participants would already be present in a clinical setting for these medical events. Pre-delivery consenting was permanently switched to virtual (electronic signed consent was obtained via DocuSign®), and visits for URIs were suspended from March 15, 2020 to September 30, 2020.

Consenting

Due to the COVID-19 pandemic, we amended our consenting process to include an electronic component. We moved from a hardcopy PRIMERO Information Packet to an electronic version. Partnered obstetricians were also provided with an electronic copy of the PRIMERO Information Packet to help with distribution to potential participants. Stage 1 consent was modified to obviate exposure risk by providing potential participants with HIPPA-compliant electronic consent forms via DocuSign®, which they were instructed to sign and return electronically. During the signing process, recruiters were on the phone with the potential participant to field any questions. Stage 2 reaffirmation (postnatal) was administered at the bedside by a recruiter in full personal protective equipment (PPE). Despite these precautions, an in-person meeting presented an exposure risk to participants and research staff. As such, the recruitment site hospital (HIMA) implemented universal screening for SARS-CoV-2 among pregnant mothers scheduled to deliver at HIMA.

Screening of mothers and babies at HIMA

As per hospital procedure, all mothers scheduled to deliver at HIMA were screened for SARS-CoV-2 antibodies using a rapid test. If the screening was positive, a PCR-based test was performed to determine if the patient had an active infection. If the mother had an active infection, all contact with her was conducted through hospital personnel assigned to the mother’s care. The protocol at HIMA for PCR-positive women presenting for labor was to isolate them in a negative pressure room within a dedicated COVID-19 ward that was on a different floor from labor and delivery. Babies born to PCR-positive mothers were also screened for SARS-CoV-2 by PCR. Treatment of mothers and children for COVID-19 took priority over PRIMERO research procedures, and continuation of birth procedures was considered on a case-by-case basis so as not to interfere with clinical care.

Birth procedures

Collection of maternal peripheral blood and administration of the baseline questionnaire was conducted at the bedside by staff. No maternal blood was collected if a mother was PCR-positive for SARS-CoV-2. If a newborn was anticipated to be infected, the child’s nasal swab for SARS-CoV-2 PCR testing was obtained immediately after birth. Results from PCR testing were available within 24 hours. If the swab was negative, PRIMERO staff proceeded as per study protocol and obtained nasal swabs. Newborn nasal swabs were obtained in a separate room by the research staff (nurse, medical technologist, or physician). Nasal swabs were not collected from infants with positive SARS-CoV-2 PCR results; however, this was an exceptionally rare occurrence, with only 1 of the 2,100 enrolled infants missing a birth nasal swab. These dyads were not excluded from the study and continued with scheduled follow-ups through remote data collection methods. In-person visits and biospecimen collection resumed once PCR testing confirmed the infant's SARS-CoV-2-negative status. This approach ensured data continuity while prioritizing safety protocols. PRIMERO staff arranged an agreement with HIMA whereby hospital staff transport the placenta from the operating room and labor suite to a separate room dedicated for PRIMERO staff to perform cord blood extractions. This minimized exposure between PRIMERO staff, participants, and hospital employees. Cord blood collection continued on all participants. Staff wore full PPE as required by HIMA and CNP guidelines and practiced appropriate social distancing when possible.

Illness surveillance visits

Clinic visits for RIs present an exposure risk to participants and research staff. Study visits that were not medically necessary (URIs in the first two years of life) were deferred during the COVID-19 pandemic from March 2020 through September 2020. Visits for URIs resumed in October 2020. Clinic visits for LRIs continued to occur regardless of study protocol because they are considered medically necessary. Research activities were conducted according to protocol after providing all necessary clinical care for each visit, with COVID-19 precautions for staff and participants as described in Supplementary Table 1. If a participant is suspected of having or confirmed to have SARS-CoV-2, a separate protocol is used (“Persons suspected or confirmed infected with SARS-CoV-2,” below).

Annual follow-up visits

Although it is uncertain how much the COVID-19 pandemic affected annual visits, study staff followed appropriate COVID-19 protocols in accordance with the local government and hospital regulations when conducting these visits.

Persons suspected or confirmed infected with SARS-CoV-2

PRIMERO staff avoided face-to-face interactions and sample collections from potential or current participants who were suspected or confirmed to be infected with SARS-CoV-2 (by PCR testing), regardless of whether they were symptomatic, by delaying in-person assessments by 10 days or until the participant had a negative PCR test for SARS-CoV-2. If a face-to-face encounter is required and COVID-19 is suspected, the child is referred for PCR testing and must have a negative result before being seen at CNP (SARS-CoV-2 testing is free in Puerto Rico). Practices followed in PRIMERO included deferring medically unnecessary visits; using PPE for all face-to-face encounters; if infection is suspected, referring for PCR testing and medical care; avoiding sample collection and face-to-face encounters while children are PCR-positive by delaying visits by 10 days or until the participant had a negative test; collecting illness characteristics via electronically administered illness questionnaires; resuming face-to-face encounters for medically necessary visits after a negative PCR result.
